# Impact of Mycotoxin Metabolites Deepoxy-Deoxynivalenol and Beta-Zearalenol on Bovine Preimplantation Embryo Development in the Presence of Acetonitrile

**DOI:** 10.3390/vetsci11060267

**Published:** 2024-06-11

**Authors:** J. Gačnikar, J. Mrkun, J. Babič, M. Sterniša, M. Zakošek Pipan

**Affiliations:** 1 Clinic for Reproduction and Large Animals, Veterinary Faculty, University of Ljubljana, 1000 Ljubljana, Slovenia; janko.mrkun@vf.uni-lj.si (J.M.); maja.zakosekpipan@vf.uni-lj.si (M.Z.P.); 2 Institute of Food Safety, Feed and Environment, Veterinary Faculty, University of Ljubljana, 1000 Ljubljana, Slovenia; janja.babic@vf.uni-lj.si; 3 Department of Food Science and Technology, Biotechnical Faculty, University of Ljubljana, 1000 Ljubljana, Slovenia; meta.sternisa@bf.uni-lj.si

**Keywords:** mycotoxins, humidity, fertility, in vitro production, deepoxy-deoxynivalenol, beta-zearalenol, acetonitrile

## Abstract

**Simple Summary:**

Early embryonic mortality is one of the main causes of poor reproductive health and is influenced by many factors, including mycotoxins and their metabolites in feed. When several mycotoxins are present in feed, they can have different effects and cause significant animal health problems; however, their synergistic effects have not yet been explored. We found that the blastocyst rate was affected by acetonitrile as a vehicle for the mycotoxin. When the desired mycotoxin concentrations were diluted with the lowest possible amount of acetonitrile, we found no statistical difference between blastocyst rates, although there was a trend suggesting that the combination caused poorer blastocyst development. However, a less invasive vehicle should be used to see if the combination of mycotoxins can affect blastocyst rates.

**Abstract:**

The quality of animal feed is increasingly affected by weather conditions, high humidity, and damage to grains, which have led to various mycotoxin-producing moulds. The aim of this study was to determine the effects of the combination of deepoxy-deoxynivalenol and beta-zearalenol on the development of preimplantation bovine embryos, the extent to which the presence of both mycotoxin metabolites affects the development of in vitro cultured bovine embryos, or whether the effect of both toxins enhances embryotoxicity. Ovaries were transported from the abattoir to the laboratory and, after maturation and fertilisation, zygotes were placed in an embryo culture medium (IVC) with different mycotoxin metabolite concentrations diluted in acetonitrile. It was found that the blastocyst rate of cleaved embryos was affected by 1 μL acetonitrile in 400 μL medium (0.25%) compared to the group without acetonitrile. For this reason, it was decided to use acetonitrile as a control group, and the desired mycotoxin metabolite concentrations were diluted in the lowest possible amount of acetonitrile (0.5 μL) that could be accurately added to the study groups. There was no statistical difference when the higher mycotoxin metabolite concentrations were added.

## 1. Introduction

Contamination of animal feed with Fusarium mould is a major problem in production worldwide. In the USA, 70% of the feed samples tested were contaminated with mycotoxins [1]. Studies at the Faculty of Veterinary Medicine, University of Ljubljana show that the most frequently detected mycotoxins are zearalenone (ZEA) and deoxynivalenol (DON) and that the occurrence of the latter has increased in recent years [2,3]. DON, ZEA, and their metabolites also pose a risk to humans when consumed in dairy products [4,5].

DON belongs to the type B trichothecenes and is found together with its acetylated metabolites (3-acetyl-DON and 15-acetyl-DON) in the species *Fusarium graminearum* and *Fusarium culmorum*. The primary toxic effect is due to the inhibition of protein synthesis. Upon contact with rumen microorganisms, DON is converted into deepoxy-deoxynivalenol (DOM-1). The deleterious effect of DOM-1 has been observed in bovine embryos produced in vitro, as development to the blastocyst stage is reduced by DOM-1 [6]. DON has toxic effects on porcine and mouse oocyte meiosis [7] and accumulates as DOM-1 in the follicular fluid of ruminants [8]. The presence of the mycotoxin metabolite DOM-1 in the follicular fluid of animals fed with contaminated feed is significant, mainly due to its affinity to accumulate in the follicular fluid itself [8]. Both DON and ZEA and their metabolites are found in urine, plasma, and cerebrospinal fluid after the feeding of contaminated feed [9]. ZEA is produced by species of the genus *Fusarium*, most commonly by *F. graminearum*, which occurs on all cereals, especially maize. It has a strong estrogenic effect and interferes with folliculogenesis and oogenesis, but not all mechanisms of action are clear. The toxin causes abnormalities during granulosa cell development and impairs follicular steroidogenesis, damages the division spindle, impairs oocyte survival, and accelerates primordial follicle activation and follicle atresia [10]. ZEA is biotransformed in the bovine gut into alpha-zearalenol (α-ZOL) (8%) and beta-zearalenol (β-ZOL) (53%) [11]. Similar to DOM-1 metabolism, the rumen and its bacteria play an important role in β-ZOL metabolism [12]. In contrast, it was found that ZEA is predominantly metabolized to α-ZOL in pigs and has a strong estrogenic effect on them [11,13,14]. The presence of the metabolite β-ZOL itself is usually detected in cattle at very low concentrations that are not considered harmful to the organism itself or to reproductive health [8,9]. In pig and mouse oocytes, ZEA prevents the elimination of the polar body [7]. We were, therefore, interested in the effects of its metabolite β-ZOL, whether there are any negative effects on the development of the bovine embryo.

The use of organic solvents is required to produce stock solutions that can be diluted in the medium of the embryo. In their work, Maes and co-workers [15] presented 14 organic solvents and carriers for screening applications in zebrafish embryos and larvae. The organic solvents that can be used in the embryo medium are acetonitrile, methanol, dimethylformamide, ethanol, glycerol, isopropanol, butanone, etc. [15]. Acetonitrile has been shown to be less compatible with their work. But, since the lowest concentration tested was 0.5%, we decided to use a concentration of less than 0.5% acetonitrile as the organic solvent for the embryo medium in our study, since the standard stock solution of mycotoxin metabolites is prepared in acetonitrile and was used in the study. Good fertility in cattle is the key to good production results. Feed is a crucial component, and the presence of mycotoxins has a major impact on the reproductive health of domestic animals [6,7,16,17]. Early embryonic mortality is the most common consequence of poor reproductive performance in dairy cattle, as the pregnancy rate on day 0 (95% heifers, 85% cows) drops by 20% to 30% (75% heifers, 55% cows) after seven days of embryonic development [18]. The aim of this study was to determine the effect of the combination of DOM-1 and β-ZOL on the development of preimplantation bovine embryos, the extent to which the presence of both mycotoxin metabolites impairs the development of in vitro cultured bovine embryos, or whether embryotoxicity is enhanced by the action of both toxins.

## 2. Materials and Methods

### 2.1. Chemicals and Reagents

The commercial stock solution for deepoxy-deoxynivalenol 50 µg/mL (DOM-1) (CAS 88054-24-4) and beta-zearalenol 10 µg/mL (β-ZOL) (CAS 71030-11-0), dissolved in acetonitrile, was purchased from Romer Labs (Romer Labs Inc., Tulln, Austria). They were stored in amber glass vials at –20 °C. Working solutions for individual mycotoxin metabolites were prepared by appropriately diluting the stock solution in acetonitrile (CAS 75-05-8) from Honeywell Inc. (Honeywell International, Inc., Charlotte, NC, USA) until the appropriate concentration of the mycotoxin working solution was reached for the in vitro experiment. Acetonitrile, methanol (from Honeywell, Inc., Charlotte, NC, USA), acetic acid, and ammonium acetate (from Merck, Darmstadt, Germany) were of p.a. or LC–MS grade purity. Deionized water was used, prepared by the Milli-Q system (Millipore, Bedford, MA, USA). For all in vitro production of bovine embryos, we used ready-to-use ovum pick-up media (OPU), oocyte and embryo wash (WASH), oocyte maturation (BO-IVM^TM^), semen preparation (BO-SemenPrep^TM^), fertilisation (BO-IVF^TM^), embryo culture (BO-IVC^TM^) medium, and oil for medium overlay (OIL), which were purchased from IVF Bioscience (IVF Bioscience, Falmouth, UK).

### 2.2. Oocyte Collection

The study took place from December 2022 to June 2023. Ovaries were collected on day minus one (day − 1) from carcasses of slaughtered animals at the slaughter line (Celjske mesnine d. d., Celje, Slovenia) and transported to the laboratory at a controlled temperature (30–33 °C). Within four hours after the removal of the ovaries, the oocyte-cumulus complexes were retrieved in the laboratory using an 18 G × 1 ½″ needle and a 3 mL syringe. Follicles ranging in size from 3 mm to 8 mm were punctured and mixed in the oocyte collection medium. The collected follicular fluid was examined with a stereomicroscope (Ernst Leitz, Wetzlar, Germany) and a search was made for grade 1 and 2 cumulus–oocyte complexes (COCs) [19,20]. COCs were transferred to a maturation medium (BO-IVM^TM^), where they matured for 22 to 24 h. BO-IVM was equilibrated in non-toxic Petri dishes the day before the material was transferred (ESP Culture Dish Four Well Lot No. 180406A-0495) and heated to 38.8 °C in 6% CO_2_ humidified atmosphere air. This was conducted according to the recommendations of the commercial company supplying the maturation media.

### 2.3. In Vitro Production of Bovine Embryos

The protocol developed was based on the use of commercially produced media from IVF Bioscience (IVF Bioscience, Falmouth, UK) and has been used previously in in vitro fertilisation research [21,22,23].

On day 0, only the COCs with intact cumulus cell layers and homogeneous cytoplasm were transferred to the fertilisation medium (BO-IVF^TM^). Sperm were added, and the combined COCs and sperm were incubated for 16 to 24 h. In all cases, the semen came from batches of two bulls with known good fertility (132009 GRAH HF, 111983 ANIS SB; Preska Insemination Centre, Slovenia). This avoided variations in semen quality and optimized the efficiency of fertilisation by selecting several bulls [24]. Both oocyte maturation and fertilisation took place in an environment with elevated CO_2_ (6%), temperature (38.8 °C), high humidity (99%), and high O_2_ (21%). This was conducted according to the recommendations of the commercial company supplying the culture media. On the following day, the cumulus was removed using the Denude pipette (V-DENUPET handle 2022/37 LOT 20176DH01 V-DENUPET 135 2022/37 LOT 22688VD35, Vitromed, Germany), and the assumed zygotes were randomly transferred to an embryo culture medium (BO-IVC^TM^) in an environment with low O_2_ (6%) and high CO_2_ (6%) in N_2_ (88%) [25].

Acetonitrile or mycotoxin metabolite working solution in acetonitrile was added to the embryo culture medium (BO-IVC^TM^) at the time of medium preparation.

### 2.4. Morphology and Kinetic Stage of Embryos

**Figure 1:**
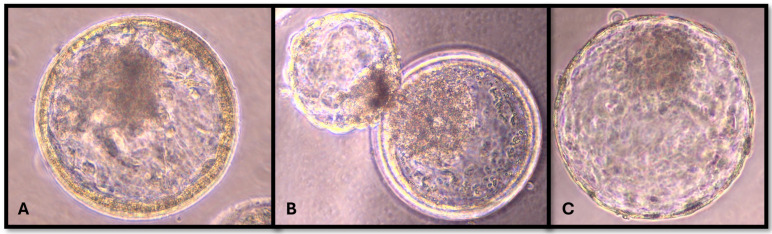
Expanded blastocyst (**A**), hatching blastocyst (**B**), and hatched blastocyst (**C**). (Author: Gačnikar J.).

**Figure 2:**
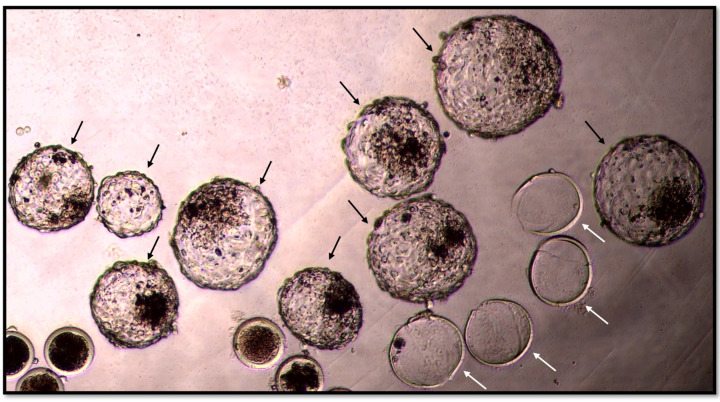
Hatched blastocysts (black arrows) and empty zona pellucida (white arrows). (Author: Gačnikar J.).

On day 8 after fertilisation, the embryos were examined by an expert in bovine embryology with an inverted microscope to determine the morphokinetic characteristics of the blastocysts and the developmental stage and quality of the embryos. This expert was blinded, as he only evaluated the embryos and was not involved in their preparation. The embryos were divided into 8 stages by kinetic classification: UFO (unfertilized oocyte/undivided embryo), 2-cell embryo, 4-cell embryo, 8–16-cell embryo, morula, blastocyst, expanding or expanded blastocyst (Figure 1), and hatching or hatched blastocyst (Figure 2). The blastocysts were classified into three grades according to Appendix 3: Consideration for evaluating in vitro-produced embryos IETS Manual 5th edition [26].

### 2.5. Determination of DOM-1 and β-ZOL Using LC/MS/MS

The measurement of DOM-1 and β-ZOL with LC-MS/MS was conducted after placing the embryos in the medium, after 8 days of development. For the LC-MS/MS determination of mycotoxin metabolites, 400 µL were used without dilution. LC/MS/MS analyses were performed on a triple-quadrupole mass spectrometer XEVO TQ equipped with an ESI interface and coupled with an Acquity UPLC system (Waters, Milford, MA, USA). Chromatographic separation was achieved on an Agilent Zorbax Eclipse RRHD Plus C18 column (2.1 ID × 100 mm, 1.7 µm) at 40 °C using gradient mode. Component A was deionized water and component B was methanol. Both components contained 0.5% acetic acid and 2.5 mM ammonium acetate. The mobile-phase flow rate was 0.3 mL/min, and the injected sample volume was 7.5 µL. MS/MS analysis was carried out in a multiple reaction monitoring (MRM) mode. During the run, the ESI source was switching between positive (ESI+) and negative (ESI–) modes. Capillary voltage in ESI+ mode was 3.4 kV, and in ESI– mode, it was 3.0 kV. ESI+ mode was used for the transition for DOM-1 (m/z 281.2 to 233.2 and 215.2) and ESI– for β-ZOL (m/z 319.2 to 159.9 and 188.1). The desolvation temperature was 500 °C, and the ion source temperature was 150 °C. Collision cell voltage and cone voltages were optimized and varied between 12 V and 40 V. The mycotoxin metabolite concentrations in the IVC medium were determined using LC-MS/MS (Waters, Milford, MA, USA), with a calibration curve with lower and higher mycotoxin metabolite concentrations (DOM-1: 50 ng/mL, 100 ng/mL, 125 ng/mL, 250 ng/mL and 400 ng/mL; β-ZOL: 0.125 ng/mL, 0.250 ng/mL, 0.375 ng/mL 0.500 ng/mL and 1 ng/mL).

### 2.6. Mycotoxin Metabolites

The concentrations of mycotoxin metabolites added to the embryo culture medium were adjusted based on previous studies on their addition to the oocytes and their presence in the follicular fluid or blood [6,7,8,27]. The correct ratio was achieved by adding the mycotoxin metabolite to the medium in the selected concentration. The embryos were kept in the same medium throughout development, as a continuous embryo culture medium was used [25]. The study was divided into an experiment with acetonitrile only and two experiments with mycotoxin metabolite (experiment 1: addition of acetonitrile only; experiment 2: addition of DOM-1 and addition of β-ZOL, and experiment 3: addition of DOM-1 and β-ZOL in combination). Each of the experiments with mycotoxin metabolites was divided into four groups (control and three experimental groups).

The volume of acetonitrile or working solution of the mycotoxin metabolite added to the 400 µL of culture medium was 0.5 µL. The same volume of acetonitrile was also added to the control groups. The working solution of the mycotoxin metabolite was prepared by diluting the stock solution of DOM-1 and β-ZOL until the appropriate concentration of the working solution was reached for the addition of 0.5 µL to the culture media. DOM-1 working solutions in acetonitrile at concentrations of 60 µg/mL, 80 µg/mL, and 100 µg/mL were prepared to achieve concentrations of 75 ng/mL, 100 ng/mL, and 125 ng/mL of DOM-1, respectively, when 0.5 µL of DOM-1 were added to 400 µL of the culture medium. β-ZOL working solutions in acetonitrile were prepared at concentrations of 96 ng/mL, 192 ng/mL, and 288 ng/mL to achieve concentrations of 0.12 ng/mL, 0.24 ng/mL, and 0.36 ng/mL of β-ZOL, when 0.5 µL of β-ZOL were added to 400 µL of culture medium. For the combination of both mycotoxin metabolites, three mixtures of working solutions containing both metabolites at appropriate concentrations were prepared to achieve suitable concentrations of 0.12 ng/mL β-ZOL and 75 ng/mL DOM-1, 0.24 ng/mL β-ZOL and 100 ng/mL DOM-1, and 0.36 ng/mL β-ZOL and 125 ng/mL DOM-1 after adding 0.5 µL of the mixture to the 400 µL culture medium. We have indeed developed an LC-MS/MS technique to measure mycotoxins and could have measured the values in stock solutions prepared with methanol. However, we decided to use acetonitrile to minimise possible inconsistencies that could arise from solvent substitution. When determining an appropriate concentration for the addition of 0.5 µL, the concentration of acetonitrile in the 400 µL culture media was 0.125% with the lowest possible effect on fertilisation for the time of the experiment.

#### 2.6.1. Experiment 1: Effect of Acetonitrile on the In Vitro Development of Bovine Blastocysts

The effect of acetonitrile on the development of bovine embryos was investigated in the first experiment. The aim was to determine the extent to which the embryos were affected or whether the effect was negligible. The assumed zygotes were randomly distributed to 400 μL cultivation and enriched with 1 μL acetonitrile and without acetonitrile in 4-well plates under the same atmospheric conditions. The blastocyst rate (blastocysts/COCs) and blastocyst rate in the cleaved embryos (blastocysts/developed embryos) were determined on day 8 after in vitro fertilisation. Four replicates of the experiments were performed.

#### 2.6.2. Experiment 2: The Effects of the Individual Mycotoxin Metabolite (β-ZOL or DOM-1 Only) on the In Vitro Development of Bovine Blastocysts

To evaluate the potential deleterious effect of β-ZOL on in vitro blastocyst development, presumptive zygotes were randomly distributed to 400 μL of cultivation media supplemented with 0 ng/mL β-ZOL (vehicle acetonitrile, control group C), 0.12 ng/mL β-ZOL (group Z1), 0.24 ng/mL β-ZOL (group Z2), or 0.36 ng/mL β-ZOL (group Z3) in 4-well plates under the same atmospheric conditions. The mycotoxin metabolite working solution in acetonitrile (0.5 μL) for each group Z (1–3) was added on day 0 to 370 μL of culture medium. On day 1, 30 μL of culture medium containing the assumed zygotes were added to the pre-warmed 370 μL drops. The blastocyst rate and the blastocyst rate in the cleaved embryos were determined on day 8 after in vitro fertilisation. Six replicates of the experiments were performed.

To determine the potential deleterious effect of DOM-1 on in vitro blastocyst development, assumed zygotes were randomly distributed in 4-well plates under the same atmospheric conditions to 400 μL of cultivation supplemented with 0 ng/mL DOM-1 (vehicle acetonitrile, control group C), 75 ng/mL DOM-1 (group D1), 100 ng/mL DOM-1 (group D2), or 125 ng/mL DOM-1 (group D3). The mycotoxin metabolite working solution in acetonitrile (0.5 μL) for each group D (1–3) was added on day 0 to 370 μL of culture medium. On day 1, 30 μL of the culture medium containing the assumed zygotes were added to the pre-warmed 370 μL drops. The blastocyst rate and the blastocyst rate in the cleaved embryos were determined on day 8 after in vitro fertilisation. Thirteen replicates of the experiments were performed.

#### 2.6.3. Experiment 3: The Combined Effect of DOM-1 and β- ZOL on the In Vitro Development of Bovine Blastocysts

To investigate the potential deleterious effect of β-ZOL and DOM-1 together on in vitro blastocyst development, presumptive zygotes were randomly distributed to 400 μL of cultivation supplemented with 0 ng/mL β-ZOL and 0 ng/mL DOM-1 (vehicle acetonitrile, control group C), 0.12 ng/mL β-ZOL and 75 ng/mL DOM-1 (group U1); 0.24 ng/mL β-ZOL and 100 ng/mL DOM-1 (group U2), or 0.36 ng/mL β-ZOL and 125 ng/mL DOM-1 (group U3) in 4-well plates under the same atmospheric conditions. The mixture of mycotoxin metabolites working solution (0.5 µL) in acetonitrile for each group U (1–3) was added on day 0 and added to 370 μL of culture medium. On day 1, 30 μL of culture medium containing the assumed zygotes were added to the pre-warmed 370 μL drops. The blastocyst rate and the blastocyst rate in the cleaved embryos were determined on day 8 after in vitro fertilisation. Sixteen replicates of the experiments were performed.

### 2.7. Statistical Analyses

The results were analysed with the Shapiro–Wilk test and compared with the analysis of variance (ANOVA). *p* < 0.05 was considered significant. The statistical analysis was performed using R 4.3.1 software. The results are presented as the mean of all blastocyst rates and for each group together with the standard error of the mean (±SEM) and are normally distributed. The experiment 1 results were analysed with SPSS 19.0 (IBM IBM Corp., Armonk, NY, USA, SPSS Statistics). We used the z-test to compare the blastocyst rate between groups of embryos [28]. The results are presented as the mean of all blastocyst rates and for each group together with the standard error of the mean (±SEM) and are normally distributed. The data on the blastocyst rates were arcsine transformed before analysis.

## 3. Results

### 3.1. Effects of Acetonitrile on the In Vitro Development of Bovine Blastocysts

This experiment included 160 COCs, which were divided into two groups of four replicates each. The results are presented in Table 1. We observed a statistical decrease when the embryos were exposed to 0.25% acetonitrile. The mean blastocyst rate in the non-acetonitrile group was 31% (SEM = ±5), and the mean blastocyst rate of the cleaved embryos (blastocysts/cleaved embryos) was 36% (SEM = ±10). The mean blastocyst rate in the acetonitrile group was 16% (SEM = ±4; *p* = 0.042), and the mean blastocyst rate of the cleaved embryos was 19% (SEM = ±4; *p* = 0.040).

### 3.2. The Effects of the Individual Mycotoxin Metabolite (Mycotoxin β-ZOL Only and Mycotoxin DOM-1 Only) on the In Vitro Development of Bovine Blastocysts

The β-ZOL experiment comprised 346 COCs divided into four groups of six replicates each. Table 2 shows the mean blastocyst rates for all groups, and as can be seen when β-ZOL was added, all blastocyst rates of the cleaved embryos were close to each other (from 25% to 27%). When the highest concentration of 0.36 ng/mL β-ZOL was added, the mean blastocyst rate of cleaved embryos was the same as in the control group (35%). We also found no differences between the stages of blastocyst development (Table 3). No statistical differences were observed with the addition of β-ZOL (Table 2).

The DOM-1 experiment included 1375 COCs divided into four groups with thirteen replicates each. The addition of the mycotoxin DOM-1 did not lead to any statistical differences in the mean blastocyst rate of the cleaved embryos, which decreased inversely linearly with increasing mycotoxin concentration. Thus, the greatest difference was observed between the control group, in which the blastocyst rate of cleaved embryos was 33%, and the group to which 125 ng/mL DOM-1 was added, which had a blastocyst rate of cleaved embryos of 28%. However, there are no statistical differences (Table 2). As shown in Table 2, we found no differences between the stages of blastocyst development.

### 3.3. The Combined Effect of DOM-1 and β-ZOL on the In Vitro Development of Bovine Blastocysts

**Figure 3:**
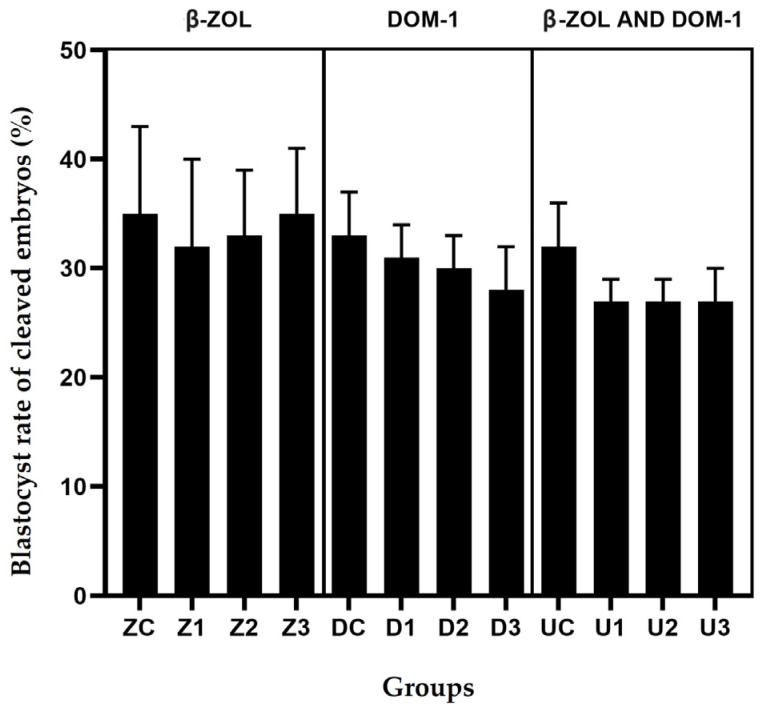
Blastocyst rate of cleaved embryos on day 8 in different concentrations of mycotoxin metabolites.

The combined experiment with β-ZOL and DOM-1 included 1558 COCs divided into four groups with sixteen replicates each. As shown in Table 2, the mean blastocyst rate of cleaved embryos was 27% in all groups U, except in the control group where it was 32% (Table 2). Despite the slight decline in the blastocyst rate shown in Figure 3, no statistical differences were observed.

The concentration of mycotoxin metabolites on day 8 of the experiment is shown in Table 3. During the experiments, the concentrations of mycotoxin metabolites in the embryo culture medium remained stable and unchanged. Only in the group with the addition of DOM-1 and in group U, in which both mycotoxin metabolites were present, was a slight decrease in the blastocyst rate observed (Figure 3). In group Z, where the concentration of β-ZOL was added, the blastocyst rate remained the same in the control group and in the groups with mycotoxin metabolites. Since we did not include the negative control group without the presence of 0.125% acetonitrile in the experiment, it is possible that the effects on the blastocyst rate in groups D and U are a combination of both concentrations, acetonitrile, and mycotoxin metabolites.

## 4. Discussion

The results of our study present the effect of a low concentration of acetonitrile and three concentration levels of the mycotoxin metabolites (DOM-1 and β-ZOL) on the 8-day bovine embryo development. The effects of acetonitrile on zebrafish and rat embryos have already been demonstrated, but we had to use it in our research design because of the solubility of mycotoxin metabolites [15,29]. Acetonitrile is one of the less well-tolerated solvents tested at early developmental stages. The lowest concentration tested, 0.5%, was completely lethal to cleavage-stage zebrafish embryos after 24 h of exposure and caused an unacceptably high level of abnormalities in 7 dpf (days post-fertilisation) larvae after 24 h of exposure [15]. It should be noted that the development of zebrafish embryos differs from that of bovine embryos. According to the literature on zebrafish, gastrulation takes place between 5.25 hpf (hours after fertilisation) and 10 hpf [30], whereas in cattle, this process occurs around day 11 [31]. In addition, the literature data show that the toxic effect of acetonitrile on zebrafish occurs at a concentration of 0.5% acetonitrile, which corresponds to 100 mM [29]. The growth of rat embryos can be affected at a concentration of 40 mM [29], but in our study, we worked with a concentration of 24 mM (0.125%) acetonitrile. However, acetonitrile could also become toxic to developing bovine embryos after day 8, which would have a detrimental effect on embryonic development. Since these data already indicated an effect of acetonitrile, we decided to use the lowest possible acetonitrile fraction (0.5 μL in 400 μL medium; 0.125%) in our groups. The results in Table 2 show that blastocyst formation is possible in the experiments on the cultivation of bovine embryos with 0.125% acetonitrile. As we could conclude that 0.125% acetonitrile can be used in the first stages of embryo development, the data from the literature [15,29] show that other solvents are more appropriate for embryo development, since the toxicity and the possibility of incidences of morphologically abnormal embryos is lower when using less toxic organic solvent in the embryo medium. Furthermore, some of the literature [6,15] mentions methanol as a most suitable solvent. In the future, more studies should be conducted with the usage of less-toxic alternatives of acetonitrile to compare the effects of methanol on bovine embryos with the results obtained in this study using acetonitrile. Previous research typically involves evaporating acetonitrile and reconstituting it with methanol to preserve cell viability, an approach that could be incorporated into future experiments.

Nevertheless, since we did not find any statistically significant differences when adding 0.36 ng/mL β-ZOL, we did not add a higher concentration of the mycotoxin metabolite and assumed that it had no effect on the development of the bovine embryo at the concentrations we added. ZEA is mainly biotransformed in the intestines of cattle into β-ZOL, which is less estrogenic than ZEA but was selected because it is present in the vast majority of cattle metabolisms in case of intoxication [11]. Based on studies, we used β-ZOL concentrations in our experiment that are comparable to the concentrations used in studies conducted after feeding mycotoxin-contaminated feed (0.02 to 0.66 mg per kg DM) [8]. Our concentrations confirmed no effects on embryonic development up to the blastocyst stage, as the final proportion of blastocysts was similar in all groups. Due to its estrogenic effect, ZEA probably has a greater negative impact on the cow uterus than on the development of the embryo itself, as these concentrations had no effect on embryo development. Larger in vitro and in vivo studies on early embryonic development would be required, including the determination of the effects of all ZEA metabolites on the embryo itself and on the cow’s reproductive organs.

The slow decline in the average blastocyst rate in the cleaved embryos when adding DOM-1 is shown in Table 2. Although there were some differences between the control group and the group with the highest mycotoxin concentration, these differences were not statistically significant. A characteristic developmental arrest was demonstrated by the researchers when the oocytes were exposed to a concentration of 100 ng/mL DOM-1. In their study, the mycotoxin metabolites were added directly to the follicle in vivo and led to various disorders of oocyte maturation. However, they found a reduction in the rate of blastocyst formation when DOM-1 was added during in vitro fertilisation [6]. Since we could not detect any differences in our study, there may be a defence mechanism in the embryos that prevents the deleterious effect of DOM-1 [32]. Since we used acetonitrile in our experiments, the results cannot be compared with the study in which the effect of mycotoxins was found in the experiments with methanol, which is less toxic for embryonic development [15,29]. In order to be able to compare the results, further studies should be carried out with less-toxic organic solvents, such as the most commonly used, methanol. Early embryonic development is crucial for a successful pregnancy and can be the most delicate part of pregnancy. In the first seven days, the pregnancy rate is 55% in cows and 75% in heifers [18]. Climatic changes have increased the incidence of mycotoxin contamination in our region and worldwide. Studies show that cattle are a species that is less susceptible to mycotoxin metabolites than others [33]. A major problem is when feed is contaminated, as more mycotoxins are formed in the presence of moulds. EFSA’s guidance is based on the presence of a single mycotoxin and does not consider the presence of multiple mycotoxins and their possible combined effect. The concentrations used in our study may not fully reflect the values found in vivo. Nevertheless, they were chosen based on previous studies, suggesting that they are very similar to the concentrations found in follicular fluid or in the oviduct. The most interesting results were obtained in the last experiment where both mycotoxin metabolites were added. Since both mycotoxin metabolites are produced by the same mould species, both are frequently found in contaminated feed [2]. In contrast to the groups in which only one toxin was added, the mean blastocyst rate of cleaved embryos immediately dropped to the lowest value when both toxins were added and remained there even with increasing concentrations of DOM-1 and β-ZOL. Nevertheless, the results between the individual U groups and the control group are not statistically significant, but the rapid decrease is of interest for further studies.

Different concentrations of β-ZOL did not change the proportion of blastocysts. However, when DOM-1 was added, the decrease was inversely proportional to the increase in concentration, and when both mycotoxin metabolites were added, the proportion of blastocysts decreased sharply even at low concentrations but without a statistically significant change. This trend remained unchanged at higher concentrations (Figure 3). The influence of the mycotoxin DOM-1 on in vitro maturation and in vitro fertilisation has already been demonstrated [6], but the zona pellucida changes during the process of maturation and fertilisation [34]. So, further studies would be necessary to determine whether it has a greater protective function during embryonic development than before fertilisation. We, therefore, assume that the results would have been different if we had decided to perforate or thin out the zona pellucida with a laser [35]. A different development of embryos with a thinned zona pellucida would show the importance of the zona pellucida as a protective layer against external influences. In our study, we did not find statistically significant changes when mycotoxins were added during embryo culture, but the results suggest that further investigation is needed, as with other solvents. Although we could not detect any differences in embryo kinetic development, the control group in experiment 3 had the highest number of blastocysts, twice as many as in groups U2 and U3 (Table 3), while the number of expanded and hatched blastocysts was close to each other. However, we were unable to detect any statistically significant changes. However, it is important to pay attention to the study of the different developmental stages of blastocysts, as this could serve as an indicator of the possible effects of mycotoxin metabolites on bovine embryos. This could indicate that only the best embryos develop in the presence of mycotoxins, while the others are inhibited in their development by the presence of mycotoxins.

## 5. Conclusions

The presence of acetonitrile at a concentration of 0.25% in the culture medium impaired the capacity of bovine zygotes to achieve the blastocysts stage. Under the conditions of the present experiment, DOM-1 and β-ZOL did not affect the formation of cattle embryos in vitro, likely due to the inclusion of 0.125% acetonitrile as a solvent. Future experiments should be conducted with other solvents with lower toxic effects compared to the acetonitrile. The synergistic effects of mycotoxin metabolites are still largely unexplored, and further research in this direction would be necessary to determine the joint effects of different mycotoxin metabolites on early embryonic development in cattle. Our study showed no statistically significant differences, despite the high number of embryos in experiment 3 (C-396, U1-381, U2-388, and U3-393). The reduction in the number of blastocysts achieved by the combination of the two mycotoxin metabolites was not statistically significant. Further studies based on a possible joint effect of the two mycotoxins’ metabolites that increase the harmful effect need to be conducted. The studies should be conducted in larger IVF centres, with the possibility of adding lower amounts of the solutions to determine the threshold for the harmful effect of the mycotoxin metabolites on embryonic development.

## Figures and Tables

**Table 1:** Results on the effect of acetonitrile on the development of bovine embryos.

Production Variable	Control	Acetonitrile Group ^e^
Cleavage rate	87.50% (70/80)	83.80% (67/80)
Blastocysts	11.43% (8/70)	4.48% (3/67)
Expanded blastocysts	11.43% (8/70)	8.96% (6/67)
Hatching or hatched blastocysts	12.86% (9/70)	2.99% (2/67)
Total blastocysts	31% ^a^ ± 5.1 (25/80)	16% ^b^ ± 4.2 (13/80)
Total blastocysts/cleaved	36% ^c^ ± 9.7 (25/70)	19% ^d^ ± 3.9 (13/67)

^a–d^ Mean values in the same line with different superscript letters differ significantly from each other (^a,b^ *p* = 0.040; ^c,d^ *p* = 0.042). ^e^ 1 μL acetonitrile in 400 μL IVC sample (0.25% of acetonitrile).

**Table 2:** Blastocyst rate and blastocyst rate of cleaved embryos on day 8 with different concentrations of mycotoxin.

Groups	Conc. ± SEM of Mycotoxin Metabolites on Day 8 (ng/mL)	Blastocyst Rate	Blastocyst Rate of Cleaved Embryos
		Proportion ± SEM (N)	Proportion ± SEM (N)
β-ZOL			
CONTROL GROUP ^1^	-	26% ± 5.7 (23/89)	35% ± 7.9 (23/65)
Z1 ^2^	0.125 ± 0.003	25% ± 6.8 (21/85)	32% ± 8.3 (21/65)
Z2 ^3^	0.245 ± 0.006	26% ± 5.2 (21/82)	33% ± 6.2 (21/63)
Z3 ^4^	0.370 ± 0.008	27% ± 5.3 (24/90)	35% ± 6.1 (24/68)
DOM-1			
CONTROL GROUP ^1^	-	25% ± 2.8 (86/340)	33% ± 3.5 (86/260)
D1 ^5^	76.5 ± 4.3	24% ± 2.7 (83/344)	31% ± 3.4 (83/266)
D2 ^6^	99.7 ± 5.5	24% ± 2.5 (81/343)	30% ± 3.1 (81/269)
D3 ^7^	126.2 ± 6.8	21% ± 3.4 (74/348)	28% ± 4.2 (74/265)
β-ZOL AND DOM-1			
CONTROL GROUP ^1^	-	24% ± 3.2 (95/396)	32% ± 3.8 (95/295)
U1 ^8^	DOM1: 76.0 ± 2.5	20% ± 1.7 (77/381)	27% ± 2.1 (77/285)
	β-ZOL: 0.126 ± 0.005		
U2 ^9^	DOM1: 99.1 ± 3.2	19% ± 1.8 (75/388)	27% ± 2.2 (75/279)
	β-ZOL: 0.246 ± 0.003		
U3 ^10^	DOM1: 125.4 ± 4.0	21% ± 2.6 (83/393)	27% ± 2.9 (83/304)
	β-ZOL: 0.382 ± 0.02		

^1^ added acetonitrile 0.5 μL (0.125%) in 400 μL IVC sample; ^2^ 0.12 ng/mL β-ZOL in 400 μL IVC sample; ^3^ 0.24 ng/mL β-ZOL in 400 μL IVC sample; ^4^ 0.36 ng/mL β-ZOL in 400 μL IVC sample; ^5^ 75 ng/mL DOM-1 in 400 μL IVC sample; ^6^ 100 ng/mL DOM-1 in 400 μL IVC sample; ^7^ 125 ng/mL DOM-1 in 400 μL IVC sample; ^8^ 0.12 ng/mL β-ZOL and 75 ng/mL DOM-1 in 400 μL IVC sample; ^9^ 0.24 ng/mL β-ZOL and 100 ng/mL DOM-1 in 400 μL IVC sample; ^10^ 0.36 ng/mL β-ZOL and 125 ng/mL DOM-1 in 400 μL IVC sample.

**Table 3:** Mean of blastocysts at different stages on the number of oocytes in groups.

Group	Blastocyst	Expanded/Expanding Blastocyst	Hatched/Hatching Blastocyst
β-ZOL			
CONTROL GROUP ^1^	8% ± 2.7	7% ± 3.2	8% ± 3.5
Z1 ^2^	10% ± 2.7	3% ± 2.2	12% ± 4.8
Z2 ^3^	7% ± 2.4	9% ± 3.5	6% ± 2.2
Z3 ^4^	6% ± 2.1	8% ± 2.3	10% ± 3.6
DOM-1			
CONTROL GROUP ^1^	6% ± 1.4	7% ± 1.7	11% ± 2.1
D1 ^5^	5% ± 1.1	13% ± 2.0	6% ± 1.2
D2 ^6^	6% ± 1.7	10% ± 1.8	7% ± 1.4
D3 ^7^	5% ± 1.4	7% ± 1.6	8% ± 1.5
β-ZOL AND DOM-1			
CONTROL GROUP ^1^	9% ± 2.2	10% ± 2.0	6% ± 1.4
U1 ^8^	6% ± 1.1	8% ± 1.6	7% ± 1.1
U2 ^9^	5% ± 1.0	9% ± 1.4	6% ± 1.7
U3 ^10^	4% ± 1.0	8% ± 1.8	9% ± 1.2

^1^ added acetonitrile 0.5 μL (0.125%) in 400 μL IVC sample; ^2^ 0.12 ng/mL β-ZOL in 400 μL IVC sample; ^3^ 0.24 ng/mL β-ZOL in 400 μL IVC sample; ^4^ 0.36 ng/mL β-ZOL in 400 μL IVC sample; ^5^ 75 ng/mL DOM-1 in 400 μL IVC sample; ^6^ 100 ng/mL DOM-1 in 400 μL IVC sample; ^7^ 125 ng/mL DOM-1 in 400 μL IVC sample; ^8^ 0.12 ng/mL β-ZOL and 75 ng/mL DOM-1 in 400 μL IVC sample; ^9^ 0.24 ng/mL β-ZOL and 100 ng/mL DOM-1 in 400 μL IVC sample; ^10^ 0.36 ng/mL β-ZOL and 125 ng/mL DOM-1 in 400 μL IVC sample.

## Data Availability

The data are contained within this article.

## References

[1] Knutsen H.K., Alexander J., Barregård L., Bignami M., Brüschweiler B., Ceccatelli S., Cottrill B., Dinovi M., Grasl-Kraupp B., EFSA Panel on Contaminants in the Food Chain (CONTAM) (2017). Risks to human and animal health related to the presence of deoxynivalenol and its acetylated and modified forms in food and feed. EFSA J..

[2] Jakovac-Strajn B., Vengušt A., Ujčič-Vrhovnik I., Pavšič-Vrtač K., Tavčar-Kalcher G. (2010). The natural occurrence of toxigenic moulds and mycotoxins in Slovenian primary grain production. Acta Agric. Slov..

[3] Babič J., Tavčar-Kalcher G., Celar F.A., Kos K., Knific T., Jakovac-Strajn B. (2021). Occurrence of Alternaria and Other Toxins in Cereal Grains Intended for Animal Feeding Collected in Slovenia: A Three-Year Study. Toxins.

[4] Torović L. (2018). Fusarium toxins in corn food products: A survey of the Serbian retail market. Food Addit. Contam. Part A.

[5] Viegas S., Assunção R., Twarużek M., Kosicki R., Grajewski J., Viegas C. (2020). Mycotoxins feed contamination in a dairy farm—Potential implications for milk contamination and workers’ exposure in a One Health approach. J. Sci. Food Agric..

[6] Guerrero-Netro H.M., Barreta M.H., Costa E., Goetten A., Dupras R., Mills LKoch J., Portela V.M., Price C.A., Chorfi Y. (2020). Effects of the mycotoxin metabolite de-epoxy-deoxynivalenol (DOM-1) on embryo development and sperm motility in cattle. J. Appl. Toxicol..

[7] Lu Y., Zhang Y., Liu J.Q., Zou P., Jia L., Su Y.T., Sun Y.R., Sun S.C. (2018). Comparison of the toxic effects of different mycotoxins on porcine and mouse oocyte meiosis. PeerJ.

[8] Winkler J., Kersten S., Meyer U., Stinshoff H., Locher L., Rehage J., Wrenzycki C., Engelhardt U.H., Dänicke S. (2015). Diagnostic opportunities for evaluation of the exposure of dairy cows to the mycotoxins deoxynivalenol (DON) and zearalenone (ZEN): Reliability of blood plasma, bile and follicular fluid as indicators. J. Anim. Physiol. Anim. Nutr..

[9] Winkler J., Gödde J., Meyer U., Frahm J., Westendarp H., Dänicke S. (2016). Fusarium toxin-contaminated maize in diets of growing bulls: Effects on performance, slaughtering characteristics, and transfer into physiological liquids. Mycotoxin Res..

[10] Zhang G.L., Feng Y.L., Song J.L., Zhou X.S. (2018). Zearalenone: A Mycotoxin with Different Toxic Effect in Domestic and Laboratory Animals’ Granulosa Cells. Front. Genet..

[11] Keller L., Abrunhosa L., Keller K., Rosa C.A., Cavaglieri L., Venâncio A. (2015). Zearalenone and Its Derivatives α-Zearalenol and β-Zearalenol Decontamination by Saccharomyces cerevisiae Strains Isolated from Bovine Forage. Toxins.

[12] Hartinger T., Kröger I., Neubauer V., Faas J., Doupovec B., Schatzmayr D., Zebeli Q. (2023). Zearalenone and Its Emerging Metabolites Promptly Affect the Rumen Microbiota in Holstein Cows Fed a Forage-Rich Diet. Toxins.

[13] Malekinejad H., Maas-Bakker R.F., Fink-Gremmels J. (2005). Bioactivation of zearalenone by porcine hepatic biotransformation. Vet. Res..

[14] EFSA Panel on Contaminants in the Food Chain (2011). Scientific Opinion on the risks for public health related to the presence of zearalenone in food. EFSA J..

[15] Maes J., Verlooy L., Buenafe O.E., de Witte P.A.M., Esguerra C.V., Crawford A.D. (2012). Evaluation of 14 Organic Solvents and Carriers for Screening Applications in Zebrafish Embryos and Larvae. PLoS ONE.

[16] Alm H., Greising T., Brüssow K.P., Torner H., Tiemann U. (2002). The influence of the mycotoxins deoxynivalenol and zearalenol on in vitro maturation of pig oocytes and in vitro culture of pig zygotes. Toxicol. In Vitro.

[17] Sangsritavong S., Combs D.K., Sartori R., Armentano L.E., Wiltbank M.C. (2002). High feed intake increases liver blood flow and metabolism of progesterone and estradiol-17beta in dairy cattle. J. Dairy Sci..

[18] Walsh S.W., Williams E.J., Evans A.C.O. (2011). A review of the causes of poor fertility in high milk producing dairy cows. Anim. Reprod. Sci..

[19] Leibfried L., First N.L. (1979). Characterization of bovine follicular oocytes and their ability to mature in vitro. J. Anim. Sci..

[20] Aguila L., Treulen F., Therrien J., Felmer R., Valdivia M., Smith L.C. (2020). Oocyte Selection for In Vitro Embryo Production in Bovine Species: Noninvasive Approaches for New Challenges of Oocyte Competence. Animals.

[21] van der Weijden V.A., Chen S., Bauersachs S., Ulbrich S.E., Schoen J. (2017). Gene expression of bovine embryos developing at the air-liquid interface on oviductal epithelial cells (ALI-BOEC). Reprod. Biol. Endocrinol..

[22] Razza E.M., Pedersen H.S., Stroebech L., Fontes P.K., Kadarmideen H.N., Callesen H., Pihl M., Nogueira M.F.G., Hyttel P. (2019). Simulated physiological oocyte maturation has side effects on bovine oocytes and embryos. J. Assist. Reprod. Genet..

[23] Shahzad Q., Pu L., Ahmed Wadood A., Waqas M., Xie L., Shekhar Pareek C., Xu H., Liang X., Lu Y. (2020). Proteomics Analysis Reveals that Warburg Effect along with Modification in Lipid Metabolism Improves In Vitro Embryo Development under Low Oxygen. Int. J. Mol. Sci..

[24] Fields S.D., Hansen P.J., Ealy A.D. (2011). Fibroblast growth factor requirements for in vitro development of bovine embryos. Theriogenology.

[25] Nagao Y., Saeki K., Hoshi M., Kainuma H. (1994). Effects of oxygen concentration and oviductal epithelial tissue on the development of in vitro matured and fertilized bovine oocytes cultured in protein-free medium. Theriogenology.

[26] Barfield J., Demetrio D. (2022). Considerations for Evaluating In Vitro-Produced Bovine Embryos—IETS Manual.

[27] Cortinovis C., Caloni F., Schreiber N.B., Spicer L.J. (2014). Effects of fumonisin B1 alone and combined with deoxynivalenol or zearalenone on porcine granulosa cell proliferation and steroid production. Theriogenology.

[28] (2013). SigmaPlot.

[29] Saillenfait A.M., Sabaté J.P. (2000). Comparative Developmental Toxicities of Aliphatic Nitriles: In Vivo and in Vitro Observations. Toxicol. Appl. Pharmacol..

[30] Kimmel C.B., Ballard W.W., Kimmel S.R., Ullmann B., Schilling T.F. (1995). Stages of embryonic development of the zebrafish. Dev. Dyn..

[31] van Leeuwen J., Berg D.K., Pfeffer P.L. (2015). Morphological and Gene Expression Changes in Cattle Embryos from Hatched Blastocyst to Early Gastrulation Stages after Transfer of In Vitro Produced Embryos. PLoS ONE.

[32] Hamdoun A., Epel D. (2007). Embryo stability and vulnerability in an always changing world. Proc. Natl. Acad. Sci. USA.

[33] Fink-Gremmels J. (2008). Mycotoxins in cattle feeds and carry-over to dairy milk: A review. Food Addit. Contam. Part A.

[34] Moros-Nicolás C., Chevret P., Jiménez-Movilla M., Algarra B., Cots-Rodríguez P., González-Brusi L., Avilés M., Izquierdo-Rico M.J. (2021). New Insights into the Mammalian Egg Zona Pellucida. Int. J. Mol. Sci..

[35] Wang Y., Chen C., Liang J., Fan L., Liu D., Zhang X., Liu F. (2022). A comparison of the clinical effects of thinning and drilling on laser-assisted hatching. Lasers Med. Sci..

